# Situating dissemination and implementation sciences within and across the translational research spectrum

**DOI:** 10.1017/cts.2019.392

**Published:** 2019-07-29

**Authors:** Aaron L. Leppin, Jane E. Mahoney, Kathleen R. Stevens, Stephen J. Bartels, Laura-Mae Baldwin, Rowena J. Dolor, Enola K. Proctor, Linda Scholl, Justin B. Moore, Ana A. Baumann, Catherine L. Rohweder, Joan Luby, Paul Meissner

**Affiliations:** 1 Department of Health Sciences Research, Mayo Clinic, Mayo Clinic Center for Clinical and Translational Science, Rochester, MN, USA; 2 Division of Geriatrics, University of Wisconsin School of Medicine and Public Health, Madison, WI, USA; 3 Department of Nursing, University of Texas Health Science Center-San Antonio, San Antonio, TX, USA; 4 The Mongan Institute, Massachusetts General Hospital, Boston, MA, USA; 5 Department of Family Medicine, University of Washington, Seattle, WA, USA; 6 Department of Medicine, Duke University, Durham, NC, USA; 7 Brown School of Social Work, Washington University in St. Louis, St. Louis, MO, USA; 8 Institute for Clinical and Translational Research, University of Wisconsin-Madison, Madison, WI, USA; 9 Department of Family and Community Medicine, Wake Forest University, Wake Forest School of Medicine, Winston-Salem, NC, USA; 10 Gillings School of Public Health, University of North Carolina at Chapel Hill, Chapel Hill, NC, USA; 11 Department of Psychiatry, Washington University School of Medicine, St. Louis, MO, USA; 12 Department of Family and Social Medicine, Albert Einstein College of Medicine/Montefiore Medical Center, Bronx, NY, USA

**Keywords:** Dissemination, implementation, translation, translational science, knowledge transfer

## Abstract

The efficient and effective movement of research into practice is acknowledged as crucial to improving population health and assuring return on investment in healthcare research. The National Center for Advancing Translational Science which sponsors Clinical and Translational Science Awards (CTSA) recognizes that dissemination and implementation (D&I) sciences have matured over the last 15 years and are central to its goals to shift academic health institutions to better align with this reality. In 2016, the CTSA Collaboration and Engagement Domain Task Force chartered a D&I Science Workgroup to explore the role of D&I sciences across the translational research spectrum. This special communication discusses the conceptual distinctions and purposes of dissemination, implementation, and translational sciences. We propose an integrated framework and provide real-world examples for articulating the role of D&I sciences within and across all of the translational research spectrum. The framework’s major proposition is that it situates D&I sciences as targeted “sub-sciences” of translational science to be used by CTSAs, and others, to identify and investigate coherent strategies for more routinely and proactively accelerating research translation. The framework highlights the importance of D&I thought leaders in extending D&I principles to all research stages.

Translational research means different things to different people, but it seems important to almost everyone.

—*Steven Woolf* (Woolf, 2008)

## Introduction

The National Center for Advancing Translational Science (NCATS) was established in 2012 with the goal of hastening the scientific process required to develop and deliver treatments that improve people’s lives. Its purpose is to advance understanding and routine use of translational science; that is, the science concerned with the process of “translation.” Translation has been defined as the problem-oriented practical process of “turning observations in the laboratory, clinic, and community into interventions that improve the health of individuals and the public” [1]. Whereas traditional conceptualizations of science are primarily concerned with the creation of new knowledge, translational science is ultimately concerned with the process of solving – through application of research knowledge – health-related problems. Thus, translational science seeks to understand the “scientific and operational principles” underlying each step of the translational process [1].

To operationalize the advancement of translational science, the Centers for Translational Science Awards (CTSA) Program has funded – since 2006 – the activities of a consortium of more than 60 academic medical centers, referred to as CTSAs, or “hubs” [2,3]. Within and across these CTSA hubs, investigators and clinicians at all stages of the translational research spectrum work together to develop, demonstrate, and disseminate strategies for overcoming common barriers to efficient and effective translation.

Ultimately, the aim of translational science is to identify guiding principles for improving the efficiency and effectiveness of research translation. One can imagine several ways in which insights gained from the sciences of dissemination and implementation (D&I) could be adapted to support this aim and enhance translation [4]. For this reason, in 2016, the CTSA consortium’s Collaboration and Engagement Domain Task Force chartered a dissemination, implementation, and knowledge translation workgroup. This special communication is a product of that workgroup.

## Objective

**Fig. 1.**
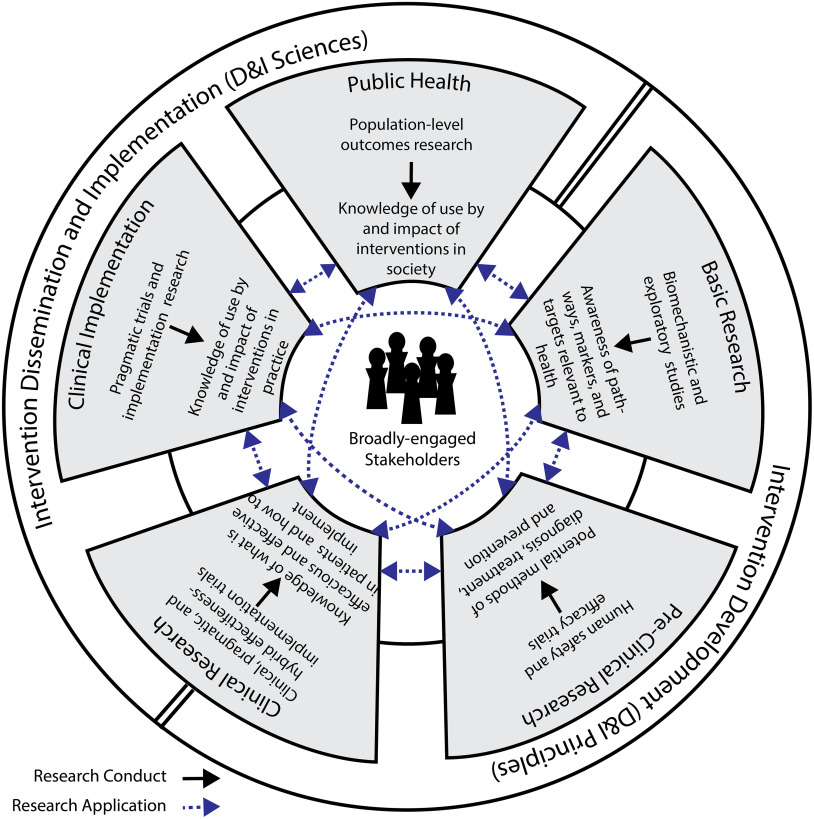
The Integrative Framework of Dissemination, Implementation, and Translation (IFDIT).

Specifically, the objectives of our workgroup were to (1) communicate the ways in which the sciences of dissemination, implementation, and translation relate and (2) explore the role of CTSAs in supporting and advancing these interrelated sciences. In pursuing these objectives, we determined that a new framework would be helpful in promoting synergy among the sciences and ensuring that the strengths of each are used to advance the work of CTSAs. As such, we also introduce the Integrative Framework of Dissemination, Implementation and Translation (IFDIT) (Fig. 1) as a guide CTSA hubs can use to support their individual and collective efforts to advance translation and improve population health.

## The Three Sciences: Dissemination, Implementation, and Translational

The National Institutes of Health (NIH) defines *dissemination* as the active and targeted distribution of information and intervention materials to a specific public health or clinical practice audience [4]. *Dissemination research* is defined as the scientific study of this phenomenon, and its goal is to expand our understanding of how best to spread the knowledge required to adopt and deploy evidence-based interventions [5]. Contemporary investigators conducting dissemination research aim to identify approaches to packaging and conveying information to improve clinical care, community, and public health services [6,7].

NIH defines *implementation* as the adoption and integration of evidence-based health interventions into clinical and community settings for the purposes of improving care delivery and efficiency, patient outcomes, and individual and population health [5]. *Implementation research* is the scientific study of this process, and its goal is to develop a knowledge base about “how” interventions become normalized and embedded within real-world practice settings and patient populations [5].

D&I research is informed and guided by underlying *sciences* [8,9] that share a common origin. These sciences trace their roots back to at least the early 1900s and to work from European and American sociologists that described the process of social change as a diffusion of ideas and innovations among individual adopters [10]. More recently, it has become clear that dissemination and implementation are separate but related processes that often co-occur (implementation following dissemination). Some argue that the D&I sciences are in fact different perspectives of the same parent science [11,12]. In the opinion of our workgroup and NIH, the two sciences are related but distinct, with each providing unique contributions to the translational process.

As mentioned above, NCATS defines *translation* as the process of turning observations into interventions that improve health [1]. Translational *research* is focused on leveraging facilitators and overcoming specific barriers to achieve this end. Translational *science* is concerned with identifying and advancing generalizable principles that hasten research translation. Translational science as a term is newer than the D&I sciences, emerging only in the last 20 years and in response to pressures to make research more useful [13]. Its explicit focus has been on increasing the efficiency and effectiveness of moving research from one stage to the next so that useful and timely research products are made available for D&I [1].

## The Integrating Principles

To investigators with exposure to and experience with all three sciences, it is clear that the sciences of dissemination, implementation, and translation are related (see Table 1). All three are concerned with solving real-world problems that impact population health, yet all three are still sciences – focused on deriving solutions that are theoretically based, generalizable, rigorously tested, reproducible, and transferable. Moreover, as is increasingly recognized, all three sciences are bidirectional. In the case of translational science, for example, observations in practice and community settings inform the research that should be conducted in the lab and in early phase human trials and vice versa. In D&I sciences, strategies are used to hasten the adoption and use of translational research products, while insights gained from understanding the contexts of practice and community settings are also used to inform the early phase selection and crafting of adoptable and usable interventions [14]. For example, brief educational meetings and audit and feedback reports are strategies that might be used to support the late-stage adoption of a clinical decision support tool, but the tool itself was first made adoptable (in early-stage research) by developing an understanding of the clinical workflow and the needs of busy clinicians. In short, the sciences of dissemination, implementation, and translation are each consistent with the ideals and function of a learning health care system – they are tools that are used to efficiently collect and apply insight to improve health. Typically, they do this through observation and the engagement of stakeholders, and through the use of diverse, often behavioral strategies designed to influence what people (e.g. researchers in translational science, and patients and practitioners in D&I) do.

**Table 1.** Extending D&I principles to early-stage translational research

**Principles from D&I sciences**	**Implications for late-stage research**	**Rationale for role in early-stage research**
Context matters and is multi-level	Successful dissemination of research knowledge and implementation of scientific discoveries are profoundly affected by healthcare policy, organizational and community culture and climate, and attitudes and behaviors of key stakeholders	All research, including basic and pre-clinical, is conducted and translated within a complex multi-level context
It is not sufficient that evidence exists and/or a practice works	We must be equally concerned with whether evidence can be disseminated to reach key stakeholders and whether practices can be taken up and delivered in real-world settings. This compels accompanying lines of inquiry	Intervention design and development benefits from appreciation of the factors that influence later-stage adoption and implementation, such as usability, desirability, feasibility, and cost
Change happens proactively	To facilitate the adoption, spread, and sustainment of improved diagnostics, therapeutic, and service delivery interventions, strategies must be developed, used, and continually improved through scientific inquiry	Even in the early stages of research, people working in systems decide what research will be pursued and how it will be done. Strategies that influence behaviors and social relationships are relevant
Both implementation practice and implementation science are team endeavors	Implementation practice requires iterative engagement and involvement of a range of stakeholders, including patients, clinicians, administrators, researchers, and policymakers. Similarly, implementation science is inherently transdisciplinary, involving treatment developers and researchers, health services researchers, and experts from closely related fields such as industrial psychology and communications research	The bidirectional exchange of information and perspectives between stages and disciplines improves the likelihood that research products will bridge translational gaps. This is true even in early stages of research

D&I, dissemination and implementation.

In a practical sense, the sciences do this work by focusing on identifying and overcoming barriers. Traditionally, translational science has focused on barriers to intervention development while D&I sciences have focused on barriers to intervention adoption and use. These conceptualizations, while useful and accurate descriptions of present realities, likely limit understanding of the full applicability of the sciences and preclude key opportunities for synergy. For example, when translation is defined only as “the process of turning observations into interventions,” [1] there is an assumption that “improvement in health” will be achieved by developing effective interventions alone, and that D&I barriers will be overcome naturally – despite a large body of evidence showing this is not the case [15–20]. Similarly, D&I sciences, in their effort to understand how best to scale up evidence-based practices, may fail to appreciate barriers to efficiently building strong, generalizable evidence and useful and usable interventions worth disseminating and implementing, as well as the role that the applicability and quality of evidence plays in the success of D&I efforts.

Finally, all three sciences are consistent with CTSA objectives to create generalizable knowledge and to identify broadly useful practices and principles. The rationale for generating products of this type is that their application has pragmatic value. Yet, differences exist among the sciences in this regard. Specifically, only translational science has communicated an explicit role for itself within all phases (e.g. T1–T4) of the research spectrum [21]. D&I sciences, in contrast, have been mostly limited in their application to the late-stage phases of research in which a viable intervention exists. This may represent a missed opportunity to leverage knowledge from decades of D&I research on how to optimize complex, real-world processes to advance translational efforts across the research spectrum. Specifically, Table 1 shows how principles that consistently guide D&I efforts within later translational research stages can find a constructive role in basic and pre-clinical research as well. Tables 2 and 3 provide examples.

**Table 2.** Example strategies and uses of D&I to improve the translational process of research conduct within each research stage

	**IFDIT stage objective**	**Research method and output**	**Potential use of translational science**	**Potential use of dissemination science**	**Potential use of implementation science**
***Basic research***	To determine whether targets, markers, or pathways exist	Basic and pre-clinical studies that generate knowledge about new targets, markers, or pathways	Develop lab registries and open science models that share insights and avoid unnecessary duplication of efforts	Seek to understand social pressures among basic scientists that inform the research questions they pursue	Study the effect of strategies to improve routine use of efficient, best practice lab procedures among staff
***Pre-clinical research***	To determine whether target, marker, or pathway can be influenced in humans	Phase 1 trials that generate knowledge about whether interventions work in humans	Advance novel study designs that improve the efficiency of phase 1 trials	Apply marketing principles to inform participant recruitment strategies to trials	Use behavioral economic strategies to improve participant engagement and retention in trials
***Clinical research***	To determine whether interventions are effective in patients	Phase 2 and 3 trials that generate knowledge about whether interventions help patients	Develop multi-site IRBs and other infrastructures to increase study efficiency	Utilize key opinions leaders to optimize communication among study teams at multiple sites	Use audit and feedback to improve adherence to study protocols among study teams
***Clinical implementation***	To determine whether interventions can be effectively delivered in practice	Phase 4 and pragmatic trials that generate knowledge about whether interventions help patients in practice and how to implement them in these settings	Develop clinical and practice-based research networks that can respond quickly to conduct T3 research	Conduct studies to ascertain optimal strategy for disseminating evidence to improve intervention adoption and reach	Conduct studies to identify and reduce key barriers to adopting and implementing in routine practice, and to reduce disparities in implementation
***Public health***	To determine whether interventions can be effectively delivered to improve population health	Observational, outcome-based studies and implementation research that generate knowledge about whether and how interventions improve population health	Develop informatics approaches and big data practices to efficiently monitor effects of wide-spread intervention roll-out	Conduct comparative studies of different dissemination strategies to determine most cost-effective method of reaching target settings and audiences	Conduct comparative studies of different implementation strategies to determine most cost-effective method of sustainably implementing in target settings

D&I, dissemination and implementation; IFDIT, Integrative Framework of Dissemination, Implementation and Translation.

**Table 3.** Example strategies and uses of D&I to improve the translational process of research application between translational stages

	**IFDIT inter-stage gap objective**	**Potential use of translational science**	**Potential use of dissemination science**	**Potential use of implementation science**	**Potential synergizing application of all three sciences**
***Basic research to pre-clinical research***	To have knowledge about a potential therapeutic target tried in humans	Develop phased funding mechanisms to pre-emptively pair pre-clinical scientists and phase 1 trialists	Compare strategies for disseminating evidence from basic research to potentially receptive pre-clinical research centers	In designing pre-clinical research intervention for trial, consider barriers to adoption of similar interventions and modify	Team science initiatives that partner multi-disciplinary basic and clinical scientists and industry
***Pre-clinical research to clinical research***	To have knowledge about an efficacious intervention in humans tried in patients	Develop funding incentives and RFAs targeted to promising pre-clinical results that encourage clinical research uptake	Compare strategies for disseminating evidence from pre-clinical research to potentially receptive clinical research centers, funders, and other stakeholder partners	In designing clinical research intervention for trial, use design methods to iteratively consider barriers to adoption among target patients and organizations and modify	Translational research centers within departments of academic medical centers
***Clinical research to clinical implementation***	To have knowledge about an efficacious intervention in patients evaluated in practice	Provide funding incentives and reduce administrative and human subjects burdens to increase receptivity for clinical implementation research	Identify organizations that could help disseminate the intervention once proven and co-create strategies for disseminating the evidence to optimize enrollment of diverse and representative practices	In designing clinical implementation intervention for trial, engage clinical stakeholders to identify barriers to adoption and modify; identify stakeholders that can lead in adopting the intervention in target organizations	Clinical and Practice-based research networks and public health collaborations
***Clinical implementation to public health***	To have knowledge about an effective intervention in practice evaluated in population	Develop partnerships with community stakeholders, policymakers, and the private sector that can facilitate efficient roll-out	Compare strategies for disseminating clinical implementation evidence to optimize adoption by diverse and representative end users	In research design, engage stakeholders in identifying optimal strategies for implementation	Region-level collaborations with policymakers, payers, advocacy groups, health systems, and public health networks

D&I, dissemination and implementation; IFDIT, Integrative Framework of Dissemination, Implementation and Translation.

## The Integrative Framework of Dissemination, Implementation, and Translation

Finally, we present the IFDIT in Fig. 1. The IFDIT seeks to integrate and expand current understanding and practice related to the dissemination, implementation, and translational sciences in order to optimize their contributions into a cohesive framework for translational activities and research. It uses as its basis an NCATS conceptualization which represents the translational research spectrum as a set of five interconnected and non-linear circular stages connecting basic research, pre-clinical research, clinical research, clinical implementation, and public health [22] (some rights reserved: https://creativecommons.org/licenses/by/2.0/). Each stage builds upon and informs each of the others via bidirectional relationships and all the stages center on and are assumed to benefit from a commitment to broad stakeholder engagement. Upon this foundation, the IFDIT adds *a conceptualization of translation as a pair of processes that occur both within and between translational stages*. The process that occurs *within* each of the five stages is termed *research conduct*, and it consists of the practical matters of carrying out a research study and generating new knowledge (e.g., formulating a research question, collecting and analyzing data, drawing conclusions). The process that occurs *between* the stages (via the 10 connecting lines) is termed *research application*, and it consists of the practical matters of *using* this new knowledge to guide the development of interventions, conduct other research, and ultimately improve health. Conceptualizing translation in this way – as a pair of processes – is intended both to (1) support targeted efforts to overcoming specific barriers within and between particular stages (e.g. by focusing on the unique goals of that stage or gap) and to (2) clarify opportunities for advancing a generalizable science of translation (e.g. by focusing on the larger conceptual issues of the underlying process, either research conduct or application).

The framework reinforces the prevailing paradigm that health interventions are first developed and evaluated (e.g. in basic, pre-clinical, and clinical stages) and then implemented (e.g. in clinical implementation and public health stages), but it includes additional guidance on the role of the D&I sciences in both activities. Specifically, the framework’s outer ring proposes that, in early-stage research, questions of intervention viability and efficacy should be prioritized, but that D&I principles should still be applied in considering the intervention’s design, usability, desirability, and potential for dissemination – a concept referred to as “designing for D&I [14,23].” Conversely, in late-stage research, questions concerning how best to disseminate and implement interventions should be prioritized and issues related to effectiveness should only be explored pragmatically and as they relate to the new circumstances in which the intervention finds itself. The framework’s outer ring transitions from one focus to the other (e.g. from intervention development and evaluation to D&I) at the mid-point of the clinical research stage. This represents the potential for hybrid effectiveness–implementation studies that occur within this stage and simultaneously consider both topics [24].

Finally, *the IFDIT positions D&I sciences as “sub-sciences” of translational science that are used, when appropriate, to contribute to the larger goal of translation*. To that end, the framework shows how understanding from and principles commonly associated with D&I sciences *can* be used to optimize processes both within and between all stages. This is best depicted in tables. Table 2 provides examples of how D&I sciences and principles can inform the conduct of research within a translational stage. Table 3 provides examples of how D&I sciences and principles help to move research from one stage to the next. Clearly, the most important and obvious role for D&I sciences is in supporting the uptake of research knowledge from one stage to another (research application) and in the later stages of research conduct. However, as shown in the tables, D&I sciences can help improve the efficiency and quality of research conduct within any stage.

## Three Examples

To further highlight the ways in which D&I sciences can and do support translation, we provide three diverse and illustrative examples. In the first example, no actual D&I research was conducted, but rather translation was advanced through an understanding of D&I processes and an application of D&I principles. In the second example, implementation research was conducted to understand barriers to adoption and to evaluate the effectiveness of implementation strategies. In the third example, D&I principles were used to integrate parallel lines of inquiry to generate new evidence, and stakeholder engagement was used to troubleshoot late-stage translational barriers. In each example, we refer to key stages and processes from the IFDIT framework.

### Example 1: Glycomacropeptide-Based Food for Phenylketonuria

Individuals with phenylketonuria (PKU) cannot metabolize phenylalanine and thus must maintain their nutrition through a synthetic amino acid formula-based diet that smells and tastes bitter and is difficult to adhere to. Basic science research at the University of Wisconsin resulted in the isolation of glycomacropeptide (GMP) in 1999, a by-product of cheese-making with essentially no phenylalanine [25,26]. After isolation – and in an example of bidirectional research application between stages – the research team reached out to the National PKU Alliance to learn about the broader context and potential desire by patients and families for development of GMP-based foods. This activity established the value of proceeding down this line of research by confirming high stakeholder demand for the product. A multi-stakeholder “GMP for PKU Task Force” was formed at the university. This “team science” initiative was intended to shorten the time required to move from pre-clinical research to clinical implementation. A researcher in nutritional science was recruited to lead mouse and human studies of GMP safety and comparative efficacy and, after finding positive results [26], the university’s Dairy Research Center was engaged to develop palatable GMP-based food products, exemplifying understanding of the multi-level context and the need to quickly move from pre-clinical to clinical research. After successfully developing several products, the research team used pre-existing collaborative relationships with PKU patients to facilitate recruitment of participants into clinical trials, thus improving the process of study conduct during the clinical research stage [25]. To support late-stage dissemination, a foundation with ties to the university provided commercialization support and a small foods company started by a family with a child with PKU took ownership of the license in 2010. Currently, this company’s GMP-based foods make up 10% of the world market of medical foods for PKU, improving adherence and quality of life for thousands of patients with PKU.

### Example 2: Reducing Early Cardiovascular Mortality Risk in Mental Health Populations

People with serious mental illness experience one of the nation’s greatest but least well-recognized health disparities: an 11–25-year reduction in life expectancy due to mainly cardiovascular causes. Building on meta-analytic data leveraging “basic” data science and demonstrating this disparity, a community-engaged partnership was formed with researchers at Dartmouth to co-develop and evaluate the “In SHAPE” intervention, a “health mentor” program with weekly coaching sessions and monthly motivational “celebrations.” Stakeholder engagement was critical in understanding the multi-level context at this pre-clinical stage and to developing an intervention that would be successful in engaging people with mental illness and leaders of mental health provider organizations. The effectiveness of In SHAPE was established in two randomized controlled trials (RCTs) demonstrating a clinically significant reduction in cardiovascular risk [27,28]. Broad uptake of the program by the public mental health sector was limited; however, due to the fact that mental health organizations were not organized, trained, staffed, or funded to provide health promotion and prevention interventions targeting cardiovascular risk factors. To address these barriers to uptake, a statewide implementation research study was designed to evaluate the effectiveness of a “learning community” consisting of program leaders from New Hampshire’s 10 mental health centers. The implementation strategy proved effective at increasing the reach while maintaining the effectiveness of the intervention (approximately half of the participants experienced clinically significant cardiovascular risk reductions [29]). To determine the most effective and feasible way to scale the intervention more broadly, a national, randomized implementation study is currently underway within 48 mental health organizations. Half of the organizations are participating in a virtual learning collaborative and the other half are receiving individual technical assistance. By considering the diverse, multi-level contexts in which the intervention is deployed, this research is able to understand whether, why, and to what extent the intervention is adapted, and the resulting effects on uptake and effectiveness.

### Example 3: Bridging Animal and Human Research to Maximize Brain Development

Animal studies in the late 1990s demonstrated a positive effect of maternal nurturance on hippocampal growth and adaptation to stress [30]. Relationships between animal researchers and human researchers in the area of brain development prompted the conduct of observational studies in humans. Insights from these longitudinal neuroimaging studies, combined with insights from additional animal research, suggested the presence of “sensitive periods” in early childhood when the brain would be more powerfully impacted by environmental forces [31]. Independent of this research, a parallel line of clinical investigation had validated and described depression in the preschool period and resulted in the development of parent–child psychotherapies. In an example of bidirectional application of research knowledge between clinical and basic research stages, Luby and colleagues bridged these two lines of investigation by using neuroimaging and measures of brain function to assess the effects of the psychotherapy on brain development and function [32]. This enabled researches to generate evidence of effectiveness for the treatment and a rationale for broad D&I. Unfortunately, and despite widespread support within the medical center and department of psychiatry, the program met contextual and policy barriers related to reimbursement (the program was designed to be delivered in a cost-effective way by master’s-level therapists, who could not be reimbursed). In an example of stakeholder engagement and the proactive development of an effective implementation strategy, the investigator team adapted the program to be delivered broadly in the school setting and is preparing to evaluate this version of the program in an RCT. Parallel efforts are underway to educate payers and other stakeholders to support policy changes that will facilitate delivery in clinical mental health settings as well.

## Discussion

In this Special Communication, we sought to communicate the relationship between translational science and the sciences of D&I and to demonstrate a potential expanded role for D&I sciences within and across the spectrum of translational research. In pursuit of this goal, we developed a framework and provided tables and examples to clearly communicate the ways in which D&I sciences can and do support the translational process. **The major proposition of the framework is that it situates D&I sciences as essential “sub-sciences” of translational science that can be used to overcome specific barriers to the translational process.** Along these lines, the framework also describes two different and equally necessary translational processes (research conduct and research application). The assumption is that advancements in translational science will need to occur both within and between translational research stages, and that these lines of inquiry may need to distinguish themselves from one another. Additionally, the framework provides, to our knowledge, the first clear examples of the ways in which principles from D&I science can be extended to support the earliest phases of translational research. Our tables do not fully communicate the multi-directional nature of processes and instead imply an overly linear journey from basic science to population health benefit. In reality, translational processes can begin with observations at any stage, move in any direction, and skip any stage. Our framework is a representation of our own ideas and experiences and does not represent empirical findings. However, we recommend that CTSAs have an opportunity to support its testing and subsequent refinement, confirmation, or refusal. Indeed, the concepts outlined in this paper should be useful to CTSAs and NCATS alike in helping to shape a coordinated agenda for the advancement of translational science. Specifically, we recommend NCATS convene a working group to consider the implications of adopting this framework as a guiding model for the activities of Domain Task Forces across the CTSA Consortia.
