# Situating dissemination and implementation sciences within and across the translational research spectrum - ADDENDUM

**DOI:** 10.1017/cts.2020.490

**Published:** 2020-07-06

**Authors:** Aaron L. Leppin, Jane E. Mahoney, Kathleen R. Stevens, Stephen J. Bartels, Laura-Mae Baldwin, Rowena J. Dolor, Enola K. Proctor, Linda Scholl, Justin B. Moore, Ana A. Baumann, Catherine L. Rohweder, Joan Luby, Paul Meissner

In the above article by Leppin et al. [1], a funding source was missing. The following statement should have appeared at the end of the financial support section:

KRS’s contribution was made possible by CTSA grant UL 1TR002645.

The original article has been updated online to include this.
